# Marine Toxins as Pharmaceutical Treasure Troves: A Focus on Saxitoxin Derivatives from a Computational Point of View

**DOI:** 10.3390/molecules29010275

**Published:** 2024-01-04

**Authors:** Norma Flores-Holguín, Joan S. Salas-Leiva, Erick J. Núñez-Vázquez, Dariel Tovar-Ramírez, Daniel Glossman-Mitnik

**Affiliations:** 1 Centro de Investigación en Materiales Avanzados, Miguel de Cervantes 120, Complejo Industrial Chihuahua, Chihuahua 31136, Chih, Mexico; joan.salas@cimav.edu.mx (J.S.S.-L.); daniel.glossman@cimav.edu.mx (D.G.-M.); 2 Centro de Investigaciones Biológicas del Noroeste, Av. Instituto Politécnico Nacional 195, Col. Playa Palo de Santa Rita Sur, La Paz 23096, BCS, Mexico; enunez04@cibnor.mx (E.J.N.-V.); dtovar04@cibnor.mx (D.T.-R.)

**Keywords:** marine toxins, saxitoxins, computational chemistry, conceptual DFT, chemical structures, chemical reactivity properties, bioavailability scores

## Abstract

This work highlights the significant potential of marine toxins, particularly saxitoxin (STX) and its derivatives, in the exploration of novel pharmaceuticals. These toxins, produced by aquatic microorganisms and collected by bivalve mollusks and other filter-feeding organisms, offer a vast reservoir of chemical and biological diversity. They interact with sodium channels in physiological processes, affecting various functions in organisms. Exposure to these toxins can lead to symptoms ranging from tingling sensations to respiratory failure and cardiovascular shock, with STX being one of the most potent. The structural diversity of STX derivatives, categorized into carbamate, N-sulfocarbamoyl, decarbamoyl, and deoxydecarbamoyl toxins, offers potential for drug development. The research described in this work aimed to computationally characterize 18 STX derivatives, exploring their reactivity properties within marine sponges using conceptual density functional theory (CDFT) techniques. Additionally, their pharmacokinetic properties, bioavailability, and drug-likeness scores were assessed. The outcomes of this research were the chemical reactivity parameters calculated via CDFT as well as the estimated pharmacokinetic and ADME properties derived using computational tools. While they may not align directly, the integration of these distinct datasets enriches our comprehensive understanding of the compound’s properties and potential applications. Thus, this study holds promise for uncovering new pharmaceutical candidates from the considered marine toxins.

## 1. Introduction

Marine organisms hold significant potential in becoming vital resources for the exploration of novel pharmaceuticals [1,2,3]. Notably, marine toxins, which mainly include toxic compounds of dinoflagellates, diatoms, and cyanobacteria by bivalve mollusks and other filter-feeding organisms such as algae in marine environments, stand out in this regard. The chemical and biological diversity exhibited by marine toxins is vast, rendering them an exceptional reservoir for uncovering new medications [1,2,3].

Paralytic toxins (PTs) constitute a group of closely related tetrahydropurines, comprising a ”family” of at least 60 analogs, each exhibiting varying levels of relative toxicity [4]. Among these, saxitoxin (STX), the first paralytic toxin to be chemically characterized, stands out as one of the most potent. These toxins pose a significant and escalating threat to human health in numerous regions around the world [5]. Their impact is observed through human intoxication, which can potentially lead to fatalities, as well as mass die-offs of both wild and farmed marine organisms due to the consumption of contaminated shellfish [6,7,8]. The potential therapeutic uses of paralytic toxins, as well as other sodium channel-blocking toxins that act at the same site as TTXs, are currently being extensively studied. These two types of non-protein marine toxins have garnered significant attention and are among the most promising candidates for a range of applications, including pain management, anesthesia, anti-tumor treatments, and anti-convulsants. Furthermore, recent advances in pharmaceutical formulations, particularly nanoformulations, have shown promising results in various murine models, enhancing the prospects for their therapeutic use.

The classification of these toxins is typically based on their chemical structures. The presence of specific functional groups influences their affinity for the sodium channel binding site, resulting in varying levels of toxicity. These toxins interact with voltage-gated sodium channels, which play a pivotal role in various physiological processes, thereby modulating the influx of sodium ions into different cell types. Upon the ingestion of these toxins, a wide spectrum of coordinated physiological functions, ranging from locomotion to cognition, can be affected [6,7,8].

Individuals poisoned by these toxins may exhibit symptoms within approximately 30 min of exposure. These symptoms often commence with a burning or tingling sensation on the lips and face, progressively leading to complete numbness, which may extend to the fingers, toes, and extremities. An overdose of STX (with a toxic dose range in humans of 1–4 mg/person) can result in death due to respiratory failure and cardiovascular shock [9].

Recent scientific research has recognized the value of exploring marine toxins and other associated bioactive molecules as a source of potential therapeutic agents and biotechnological applications [10,11,12,13,14]. By evaluating the complex interactions between these toxins and their molecular sites of action, important insights into the cellular and physiological mechanisms of disease can be gained [1]. The potential therapeutic applications of these marine toxins encompass a large number of diseases like cancer, Alzheimer’s, diabetes, pain, AIDS, microbial diseases (fungal and bacterial), inflammation, allergy, osteoporosis, asthma, epilepsy, and schizophrenia [10,11,12]. A great diversity of marine toxins, such as tetrodotoxins, saxitoxins, gonyautoxins, gymnomidins, domoic acid, spirolids, ciguatoxins, gymnocins, protoceratins, karlotoxins, gambierols, brevetoxins, pectenotoxins, azaspiracids, lygbyatoxins, oscillatoxins, kalkitoxin microcystins, gambieric acids, gonodiomins, caribenolides, amphidinols, amphinolides, ceramide, symbioramide, palytoxin, yesotoxin, and okadaic acid, are being evaluated for these potential therapeutic uses [10,11,12].

Saxitoxin serves as the prototype for a series of compounds characterized by a fundamental trialkyl tetrahydropurine structure [15]. This structure features NH2 groups at positions 2 and 8 of the purine ring, forming the two permanent guanidinium moieties [16]. The diversity within this family of compounds arises from variations in the functional groups positioned at four different sites around the purine ring. These variations have led to the classification of these compounds into distinct divisions, which include the carbamate toxins, N-sulfocarbamoyl toxins, decarbamoyl toxins, and deoxydecarbamoyl toxins [2].

Interestingly, certain substitutions at these positions can result in a reduction in toxicity relative to saxitoxin, with the exception of GTX1, which exhibits a toxicity level comparable to that of saxitoxin [17]. Among these toxins, the most potent ones belong to the carbamate division, with the following hierarchy: saxitoxin (STX), neo saxitoxin (NeoSTX), and gonyautoxin (GTX1). Exhibiting intermediate toxicity are gonyautoxin 2, 3, and 4 (GTX2, GTX3, and GTX4, respectively), while those in the decarbamoyl toxins division (dcGTX2 and dcGTX3) are characterized by lower toxicity [18].

While previous studies have delved into the physicochemical properties of certain analogs of paralytic toxins [15,16,19,20,21], the current investigation extended beyond this by assessing the chemical reactivity and other aspects of the 18 analogs under scrutiny.

We also find it valuable to emphasize in the introduction that, given the current opioid epidemic, there is a need for potent analgesics that do not lead to addiction. Therefore, the exploration of new drugs with analgesic and anesthetic properties, including the use of sodium channel-blocking toxins such as paralytic toxins (PTs) and tetrodotoxins (TTXs), holds therapeutic potential [11,13]. Consequently, the primary objective of this research was to conduct a comprehensive computational characterization of 18 saxitoxin (STX) derivatives. The aim was to explore their potential as drug precursors by analyzing their reactivity properties within marine sponges, utilizing conceptual density functional theory (CDFT) techniques [22,23,24,25,26,27,28,29,30]. Simultaneously, this study delved into their pharmacokinetic properties, bioavailability, and druglikeness scores, which were assessed using freely available software web tools [31,32]. It is important to acknowledge that establishing a direct correlation between the ADME parameter values presented here (calculated through the very accurate SwissADME software (http://www.swissadme.ch)) and the CDFT chemical reactivity results may not be feasible.

Nevertheless, it is equally important to recognize that these two sets of findings offer complementary information. To elaborate, while ADMET parameters provide insights into various facets of a compound’s pharmacokinetics and safety profile, CDFT chemical reactivity results shed light on its chemical behavior and reactivity patterns. Consequently, while they may not align directly, the integration of these distinct datasets enriches our comprehensive understanding of the compound’s properties and potential applications.

The toxic equivalent factor (TEF) is a measure used in toxicology to assess the relative toxicity of different substances, particularly environmental pollutants like dioxins and furans. TEF values are assigned to chemicals based on their potency compared to a reference substance, typically 2,3,7,8-tetrachlorodibenzo-p-dioxin (TCDD). These factors help standardize risk assessments and regulatory decisions by considering the cumulative toxic effects of various chemicals within a mixture. Understanding the TEF is crucial for evaluating the environmental and health impacts of complex chemical exposures [21,33].

In this context, the LD50, or median lethal dose, is a measure used in toxicology to assess the lethality of a substance. It represents the dose at which 50% of a test population is expected to die. This metric helps evaluate the potential harm of various substances, aiding in regulatory decisions and safety assessments. It is crucial for understanding the toxicity levels of chemicals, drugs, or other agents, though ethical concerns surround the use of lethal doses in experiments.

For reference, the names, abbreviations, molecular formulae, molecular structures, LD50 and TEF values, and associated tags for this research can be found in Table 1a–c. The LD50 and TEF values were taken from reference works [21,33].

## 2. Results and Discussion

The systems were optimized to find the minimum energy geometry and then verified through vibrational frequency analysis. Using the optimized structures, reactivity properties were calculated prior to optimization in a water solvent. Additionally, an analysis of the vibrational frequencies was conducted to confirm that they represented the minimum energy conformations.

**Figure 1:**
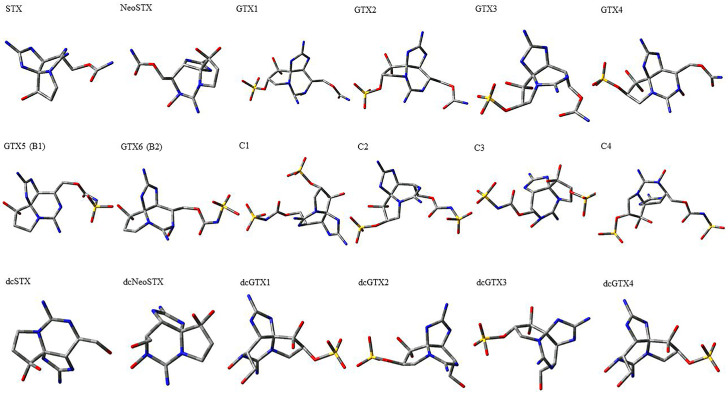
Graphical display of the optimized molecular structures of saxitoxin and its derivatives.

Utilizing the ground-state geometry as a starting point, we conducted energy calculations on the structures of saxitoxin derivatives, considering both neutral and ionic characteristics, to determine their reactivity descriptors. The outcomes of these calculations are presented in Table 2. For visual reference, the optimized structures resulting from these calculations are illustrated in Figure 1.

Table 2 reveals that the electron affinities (EAs) consistently fell below the 1 eV threshold, approaching zero in certain carbamates. The electronegativity, which represents an atom’s attraction to shared electrons, tended to be lower for carbamates, with the exception of GTX1 at 3.44 eV, ranking among the highest, only surpassed by N-sulfocarbamoyl at 3.45 eV. This suggests that carbamates readily release their electrons, with GTX2 displaying the lowest value at 2.73 eV, the most noteworthy instance for this descriptor.

Now, we consider chemical hardness η and electrophilicity ω, the former signifying a system’s ability to interact with other molecular systems. Notably, GTX2 exhibited the lowest chemical hardness at 4.29 eV, in harmony with its chemical potential value, indicating a heightened capability for interactions. In contrast, electrophilicity represents the ability to stabilize a system after saturation with electrons from the surroundings. Here, C1 stood out with a value of 1.07 eV, showcasing the highest capacity, followed closely by GTX1 at 1.04 eV. C1’s exceptional ability in this regard could be attributed to its occupied bonding sites and the presence of two saturated sulfonyl groups.

Furthermore, the electrophilicity ω index entails a trade-off between an electrophile’s inclination to gain additional electron density and its resistance to exchanging electron density with its surroundings [30]. A classification of organic compounds into strong, moderate, or marginal electrophiles, represented by an electrophilicity ω scale, was established by examining a set of Diels–Alder reactions and the respective electrophiles involved in them [34,35,36]. For the first category, electrophilicity ω exceeds 1.5 eV; for the second category it falls between 0.8 and 1.5 eV; and for the final category, ω is less than 0.8 eV [34,35,36]. Upon reviewing Table 2, it is evident that all investigated marine toxins exhibited behavior characteristic of moderate electrophiles, with the exception of GTX3, GTX4, and GTX5, which could be considered marginal electrophiles. Domingo and his collaborators [30,35,37,38,39] also introduced a nucleophilicity index N, determined by considering the HOMO energy obtained through the Kohn–Sam (KS) scheme with an arbitrary shift of the origin, using the molecule of tetracyanoethylene (TCE) as a reference. Their analysis of various nucleophilic species involved in polar organic reactions led to a classification of organic molecules as strong nucleophiles (N > 3.0 eV), moderate nucleophiles (2.0 < N < 3.0 eV), and marginal nucleophiles (N < 2.0 eV). Upon revisiting Table 2, it can be concluded that, except for GTX2, which qualified as a strong nucleophile, all other molecules could be categorized as moderate nucleophiles.

For this kind of molecule, it is expected that the electrodonating power $\omega^{-}$ is larger than the electroaccepting $\omega^{+}$. Indeed, this can be verified after the inspection of Table 2, with STX displaying the highest value among all the marine toxins. Slightly lower values were found for GTX1 and C1, followed by C2, C3, and C4 and dcGTX1, dcGTX2, dc GTX3, and dc-GTX4. The lowest value for this descriptor was exhibited by dc-STX. It is interesting to note that a simple structural modification was made in the transition from STX to NeoSTX to diminish the value of the electrodonating power $\omega^{-}$ for this molecule, evidencing the difference in the predicted chemical reactivities. As the values of the chemical reactivity index, denoted as the net electrophilicity ($\Delta \omega^{\pm }$), resulted from an interplay between $\omega^{-}$ and $\omega^{+}$, their predicted values should follow the tendency expressed through the consideration of both descriptors. The highest value for $\Delta \omega^{\pm }$ was displayed by C1, followed closely by GTX1, C2, C3, C4, dcGTX1, dcGTX2, dcGTX3, and dcGTX4. Finally, the smallest values of this descriptor were shown by GTX3, GTX4, and GTX5.

To calculate drug-likeness and pharmacokinetic properties, the study’s systems first had to be converted into their Simplified Molecular Input Line Entry Specification (SMILES) notations. Then, the likelihood of a molecule becoming a drug was assessed. These calculations relied on machine learning algorithms for binary classification, linear regression, and similar chemoinformatics techniques. The results of the drug-likeness properties are summarized in Table 3.

By checking the results from Table 3, it can be seen that all the studied compounds displayed log P values below 5 in accordance with the original Lipinski’s Rule of Five (RO5) recommended values [40]. Additionally, all the values were negative, indicating that all the compounds exhibited hydrophilic characteristics or possessed a higher affinity for the aqueous phase. However, it can also be noted from Table 3 that all the considered molecules displayed violations to the RO5 for other descriptors like the polar surface area (PSA), the molecular volume, and the number of rotatable bonds, as explained below. Although not shown in Table 3, it is worth mentioning that all the studied toxins violated the RO5 regarding the number of hydrogen bond donors and acceptors [40].

When considering the concept of polar surface area (PSA), it is crucial to note that the derivatives exceed the threshold of 140 ${Å}^{2}$. This elevated value signifies limited permeability through cell membranes. This descriptor proves particularly effective in predicting drug absorption, as highlighted in the study by Winiwarter et al. (1998) [41].

Among the compounds analyzed, dcSTX exhibited the lowest PSA value at 154 ${Å}^{2}$, followed by deNeoSTX at 165 ${Å}^{2}$ and dcGTX1 at 186 ${Å}^{2}$. In contrast, the remaining values hovered around the 200 mark, with some even surpassing 300 ${Å}^{2}$.

The incorporation of sulfur atoms into these derivatives carries significant importance, as it has been reported that PSA values including sulfur atoms exhibit a stronger correlation with human jejunum permeability compared to PSA values based solely on oxygen (O) and nitrogen (N) [41].

Molecular volume plays a pivotal role in determining the journey of a drug from its administration site to its target action site, as elucidated by Bartzatt in 2005 [42]. Among the compounds analyzed, the lowest volumes were observed in dcSTX and dcNeoSTX, with volumes measuring 221 and 230 ${Å}^{3}$, respectively.

On the contrary, the N-sulfocarbamoyl derivatives exhibited the highest molecular volumes. This phenomenon can be attributed to the incorporation of the sulfocarbamoyl group, known for its significant volume. Notably, this descriptor assumes great importance during the absorption phase. Drugs with larger molecular volumes might encounter obstacles when traversing cellular membranes, in contrast to their smaller counterparts. This variance in permeability can ultimately influence the drug’s bioavailability and overall effectiveness.

Regarding the RO5, it is noteworthy that all the derivatives under examination deviated from this rule. Specifically, each of them exhibited two violations, except for dcSTX and dcNeoSTX, which had only one violation each. When this descriptor is breached multiple times, it can potentially lead to complications in terms of bioavailability.

The count of rotatable bonds varied among the compounds analyzed, with distinct implications for their properties. dcSTX and dcNeoSTX stood out with just one rotatable bond each. The decarbamoyl derivatives possessed two, while STX and NeoSTX had three. Carbamates B1 and B2 exhibited four, and the number escalated to five for carbamates GTX1, GTX2, GTX3, and GTX4. Remarkably, the N-sulfocarbamoyls surpassed all of these compounds with six rotatable bonds, rendering them exceedingly flexible, perhaps excessively so for oral bioavailability considerations.

The capacity for molecular rotation plays a pivotal role in a molecule’s flexibility, potentially influencing its binding potency. Conversely, a low number of rotatable bonds can lead to rigidity within the system, affecting its chemical reactivity. These factors collectively underscore the significance of rotatable bonds in understanding the behavior and properties of these compounds.

Pharmacokinetic descriptors were acquired using the SwissADME web tool [43], and a summary of these descriptors is presented in Table 4. Upon reviewing this summary, it becomes apparent that all the saxitoxin derivatives shared a common trait: they exhibited low gastrointestinal (GI) absorption and showed no permeation through the blood–brain barrier. This observation presents a challenge for these derivatives, as the structural and physicochemical attributes of a drug molecule are undeniably pivotal factors in drug design. An issue of paramount importance revolves around the molecule’s ability to effectively and rapidly traverse various biological membranes, allowing for the accumulation of therapeutic concentrations at the intended target, as emphasized by Martin in 2005 [44].

Within the carbamate group, none of the compounds did not function as P-glycoprotein (P-gp) substrates. Additionally, both the N-sulfocarbamoyls and decarbamoyl derivatives were classified as P-gp substrates, with the exception being GTX5(B1). This categorization suggests that these compounds could potentially be transported within the body by P-gp, a protein that acts as a drug efflux pump.

Another noteworthy descriptor pertains to the negative nature of these saxitoxin derivatives as Cytochrome P450 family enzyme inhibitors. This signifies that these compounds will not hinder the metabolic activity of CYP450 enzymes, allowing them to undergo metabolic biotransformation. Of particular significance is their lack of inhibition towards CYP3A4, the most crucial liver cytochrome accounting for 60% of all hepatic cytochromes and responsible for the biotransformation of approximately 46% of commonly used drugs.

The skin permeation values observed for these derivatives ranged from −11.25 to −14.78. As mentioned previously, it is important to note that the more negative the log Kp value (expressed in cm/s), the lower the skin permeability of the molecule in question.

Utilizing the SwissADME prediction tool, an additional score that contributed to characterizing the drug-likeness of the evaluated systems was identified. This score is known as the Abbott Bioavailability Score (AAS), and its purpose is to assess the likelihood of a compound possessing at least 10% oral bioavailability in rats or measurable Caco-2 permeability, as defined by Martin in 2005 [44]. The AAS assigns values within specific ranges, namely 11%, 17%, 56%, or 85%, to the molecular systems under scrutiny.

**Figure 2:**
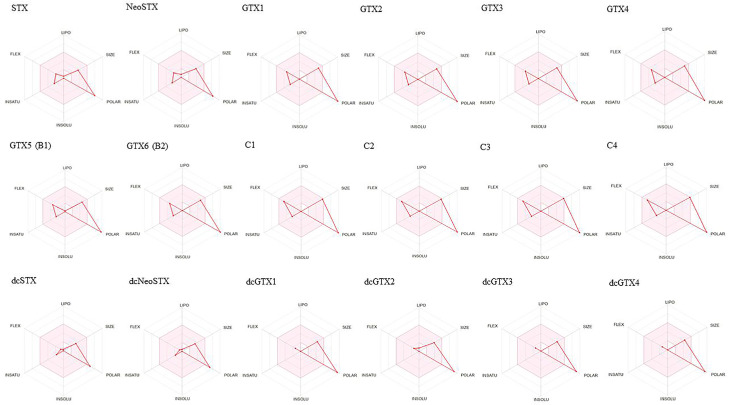
Graphical display of the bioavailability radars of saxitoxin and its derivatives.

In our analysis, all the derivatives yielded an AAS score of 0.17, except for dcSTX and dcNeoSTX, which recorded a higher score of 0.55. Furthermore, a comprehensive plot known as a bioavailability radar, depicted in Figure 2, was generated, considering six crucial properties of these systems: lipophilicity, size, polarity, solubility, flexibility, and saturation.

Ideally, a molecule should occupy the entire pink area of the bioavailability radar plot to be considered a good drug candidate. However, the results revealed a distinctive profile characterized by very high polarity, low solubility, and lipophilicity. None of the systems exhibited the ideal combination required to completely cover the pink area, indicating that none of them aligned perfectly with the criteria for ideal drug-likeness in this particular graphical evaluation.

### Clinical Applications of Paralyzing Toxins

There is an important bibliography related to the potential clinical applications of paralyzing toxins as well as other Na+ channel-blocking toxins, such as tetrodotoxin. It highlights their potential uses as analgesics and anesthetics (local) in the treatment of conditions such as acute and chronic anal fissures, reducing anal tone, and in the safe and effective treatment of chronic tension-type headaches and visceral pain associated with gastrointestinal disorders, as well as their anticonvulsant and anti-inflammatory effects [45,46,47,48,49,50,51]. In veterinary medicine, they can effectively manage bucked shin pain as local long-acting pain blockers (equine model) and block pain and inflammation in equine osteoarthritis [52,53]. Similar to the findings described by Bucciarelli et al. [12] for tetrodotoxin, it is possible that paralyzing toxins have potential therapeutic applications, particularly for the treatment of pain (cancer-related, neuropathic, and visceral). Additionally, these marine toxins may also have other applications in a wide variety of medical conditions such as heroin and cocaine addiction, spinal cord injuries, brain injuries, epilepsy, and some types of tumors.

Various investigations coincide in centrally describing the special interest in the potential therapeutic uses of paralyzing toxins and tetrodotoxin as powerful analgesics, since acute or chronic pain produced by multiple diseases affects tens, perhaps hundreds, of millions of people around the world, costing millions of dollars a year, coupled with the associated opioid epidemic. Therefore, there is significant interest in developing effective analgesics that are non-addictive, this being one of the main advantages over other drugs. Melnikova et al. [54] and Buciarelli et al. [12] conducted an extensive review of the clinical applications of tetrodotoxin and studies focused on improving the efficacy and safety of tetrodotoxin when used together with other substances and additional drug delivery systems. The use of tetrodotoxin has even been described as a therapeutic for the control and management of heroin and cocaine addiction [12,55,56]. Additionally, these toxins have been applied as anesthetics with the aim of identifying new drugs with better and longer-lasting activity [13,52,57], and they have recently been explored as potential anti-inflammatory drugs with better performance in some pathologies compared to other drugs in routine use [49,54]. A similar example of the potential therapeutic applications of these marine toxins is the development and clinical use of botulinum toxins [58,59,60].

## 3. Materials and Methods

The methodology employed by our research group involved constructing the system and searching in chemical structure databases. The 18 molecules under study were sourced from the PubChem database (https://pubchem.ncbi.nlm.nih.gov/, (accessed on 1 April 2023 )). Subsequently, a conformer search was conducted to determine the most stable shape that the molecules assumed as a result of rotation around a single bond or conformation changes. This conformer was obtained using the Marvin View 17.15 program (ChemAxon, Budapest, Hungary) with the application of Molecular Mechanics Force Fields [61,62,63,64,65].

Once the most stable shape was determined, the next step involved optimizing the geometry in a gas-phase environment. This optimization was followed by the calculation of vibrational frequencies to verify that the true minima were reached. The geometry optimization was performed using the semi-empirical PM6 method, a technique that has consistently demonstrated its reliability and precision in providing accurate geometrical results within our research group’s work [66,67].

The electronic property calculations were carried out utilizing the atomic arrangement that exhibited the highest stability, which corresponded to the lowest energy state. These calculations aimed to determine reactivity descriptors based on conceptual density functional theory. The methodology employed the Def2TZVP basis set [68,69] and the MN12SX density functional [70]. Water was used as the solvent, and the SMD solvent model [71] was incorporated into the calculations. To verify the fulfillment of the ionization energy theorem, the KID (Koopmans in DFT) procedure was applied [72,73,74,75,76]. This involved analyzing the results obtained from the optimization and frequency calculations, along with the energy calculation of the ground states. All electronic calculations were conducted using Gaussian 16 Revision C.03 software [77].

With the results of the electronic calculations, we could obtain the energy values used to calculate concepts that define the reactivity of systems, known as global descriptors. These descriptors measure the complete susceptibility of a system to various types of reactions. Firstly, there are the electron affinity (EA) and ionization potential (IP), which gauge the system’s tendency to accept or donate one electron. Using the previously established KID procedure [72,73,74,75,76], the CDFT descriptors could be expressed in terms of the energy of the frontier orbitals: highest occupied molecular orbital (HOMO) and lowest unoccupied molecular orbital (LUMO). The equations for electron affinity (EA) and ionization potential (IP) are as follows: $\epsilon_{L}$ = – EA and $\epsilon_{H}$ = – IP, where $\epsilon_{L}$ and $\epsilon_{H}$ are the corresponding energies of the LUMO and HOMO orbitals, respectively [22,23,24,25,26,27,28,29].

The electronic chemical potential, denoted as μ, represents the first derivative of the energy with respect to the number of electrons. It measures the tendency of electrons to escape from systems in equilibrium, which is equal to 0.5 ($\epsilon_{H}$ + $\epsilon_{L}$). This quantity has been established as the negative of electronegativity χ [22].

Hardness, η, a second derivative of the energy with respect to the number of electrons, quantifies a molecule’s resistance to intramolecular charge transfer, which is equal to ($\epsilon_{L}$ – $\epsilon_{H}$) [22].

Electrophilicity measures the ability of an agent to accept electrons from the environment and represents the stabilization energy when a system gains an additional electronic charge from the external environment, calculated as $\omega \approx {\left(\epsilon_{L}+\epsilon_{H}\right)}^{2}/4\left(\epsilon_{L}-\epsilon_{H}\right)$ [78,79].

The electrodonating power refers to the ability of a chemical system to donate a small fractional charge, calculated as $\omega^{-}\approx {\left(3\epsilon_{H}+\epsilon_{L}\right)}^{2}/16\eta$. The electroaccepting power, on the other hand, signifies the ability of a chemical system to accept a small fractional charge, calculated as $\omega^{+}\approx {\left(\epsilon_{H}+3\epsilon_{L}\right)}^{2}/16\eta$ [24]. Finally, the net electrophilicity $\Delta \omega^{\pm }=\omega^{+}-\left(-\omega^{-}\right)=\omega^{+}+\omega^{-}$ [25], as a comparison of the former two properties.

After determining the chemical reactivity properties, we predicted the systems’ pharmacological properties, specifically drug-likeness and pharmacokinetic properties.

Drug-likeness involves calculating various molecular descriptors to facilitate the virtual screening of large molecule collections. This process helps identify potential drug candidates while eliminating structures lacking drug-like properties. The descriptors, obtained from http://www.molinspiration.com, (accessed on 1 April 2023), include the following:

- Log P, or the octanol–water partition coefficient: This descriptor quantifies the hydrophilic or hydrophobic nature of the system, indicating how readily a moiety or analyte will partition between aqueous and organic phases [80].
- Molecular polar surface area (PSA): The PSA is a fragment-based methodology that derives standardized contributions to the molecular polar surface area from functional groups and atom types [81,82].
- Molecular volume: The molecular volume refers to the area occupied by a molecule in three-dimensional space. It is determined by fitting the sum of fragment contributions to the ”real” 3D volume for a training set of approximately twelve thousand molecules, most of which are drug-like [31,32].
- Rule of Five: This rule posits that most ”drug-like” molecules should have characteristics such as log P ≤ 5, molecular weight ≤ 500, a number of hydrogen bond acceptors ≤ 10, and a number of hydrogen bond donors ≤ 5. Molecules violating more than one of these rules may face bioavailability challenges [40].
- Number of rotatable bonds (nrotb): This topological parameter measures molecular flexibility and serves as a good descriptor for the oral bioavailability of drugs. Rotatable bonds are defined as any single non-ring bond connected to a non-terminal heavy (i.e., non-hydrogen) atom [83].

These pharmacological properties are essential for assessing the potential suitability of molecules for drug development.

Additionally, the efficiency of a drug involves reaching the target with the right concentration and bioactive features to promote the expected biological actions [83]. Pharmacokinetic properties describe how drugs pass into, through, and out of the body [84]. SwissADME, a free academic web tool, evaluates the individual absorption, distribution, metabolism, and excretion (ADME) behaviors of the molecule under investigation based on specialized models. The predictions from these models are compiled in the pharmacokinetics section [43].

The last section analyzes gastrointestinal absorption (GI), determined by the permeability of the GI mucosa and the transit rate in the GI tract [85]. The blood–brain barrier (BBB) is a shield protecting the brain, consisting of enzymatic activities and active efflux. BBB permeation is fundamental for the distribution of centrally acting molecules or, conversely, for limiting unwanted effects of peripheral drugs [86]. Permeability glycoprotein (P-gp) substrates are important proteins of the cell membrane that pumps many foreign substances out of cells. The models return either ’Yes’ or ’No’ to indicate if the molecule under investigation has a higher probability of being a substrate or a non-substrate of P-gp (respectively, an inhibitor or a non-inhibitor of a given CYP) [43]. CYP1A2, CYP2C19, CYP2C9, CYP2D6, and CYP3A4 are inhibitors of Cytochrome P450 family enzymes. These five isoenzymes are involved in the metabolism of 90% of drugs. SwissADME measures if the studied system has an inhibitory character. If the result is ’Yes’, the system blocks the metabolic activity of one or more CYP450 enzymes with two possible effects.

If the effect is on an active drug that requires biotransformation to be excreted, its clearance will be decreased, leading to the potentiation of the effect, which may reach toxic levels. In the case of prodrugs, the inhibition of their metabolism will result in a lower concentration of the active metabolite and thus a reduction in their effect [29]. SwissADME performs multiple linear regression aimed at predicting the skin permeability coefficient (Kp). This approach is adapted from Potts and Guy [87], who found that Kp is linearly correlated with molecular size and lipophilicity (R2 = 0.67). The more negative the log Kp (in cm/s), the less skin-permeant the molecule is [43].

While there is concern regarding the systematic application of such marine toxins, such as paralyzing toxins and tetrodotoxin, because their therapeutic index tends to be low (intravenous or intramuscular injection), due to their high toxicity, several studies have shown that coadministration with vasoconstrictors, local anesthetics, or oral pellets in murine models significantly increases this therapeutic index, in addition to general efficacy [12,45,88,89]. Although lethal doses for humans range between 1 and 2 mg for paralytic toxins and tetrodotoxin, when administered at levels well below the LD50 in the form of microdoses, these two types of toxins have therapeutic properties of interest. Therefore, in addition to local applications, the evaluation of various analogs with diverse toxicities (some modified); the mutagenesis of proteins, isoforms, and toxins in very low concentrations coupled with slow- and safe-release polymers; and new experimental pharmaceutical forms in the process of development, including nanoformulations, have been investigated [12,13,57,90,91]. In recent years, there has been growing interest in targeted drug delivery due to the important advantages that these systems offer over a wide range of medications. The immobilization of active compounds using nanocarriers can increase the bioavailability of marine toxins such as tetrodotoxin, improve their solubility, overcome various barriers, reduce their toxicity, and achieve sustained-release drugs. In this way, the encapsulation of tetrodotoxin has been carried out, which reduced its toxicity and increased the duration of the anesthetic effect, generating broad prospects for the development of new and effective drugs based on toxins [54].

## 4. Conclusions

The primary objective of this research was to comprehensively characterize various properties of the marine toxin saxitoxin and its 18 derivatives. We employed molecular modeling and computational chemistry techniques, with a specific focus on density functional theory for assessing chemical reactivity properties. Additionally, we utilized readily available web tools like Molinspiration and SwissADME to delineate their pharmacological attributes.

Within our study, we examined a family of 18 molecules, seeking to discern their potential suitability as effective drugs. To meet this criterion, these molecules needed to possess certain key properties. These included the ability to interact with other molecules in their environment, permeate natural cellular barriers, potentially serve as substrates for specific proteins like glycoprotein, exhibit a defined topological polar surface area (PSA), and display a level of rigidity influenced by the number of rotatable bonds. Furthermore, they had to effectively reach their target, form bonds with it, undergo metabolism, and be excreted. The molecules we scrutinized demonstrated several of these crucial parameters essential for pharmaceutical potential.

In terms of log P values below 5, all the compounds conformed to Lipinski’s RO5, and none of them exhibited a PSA lower than 140 ${Å}^{2}$, which could impede cell membrane permeation. Notably, dcSTX, with a PSA of 154 ${Å}^{2}$, and dcNeoSTX, with a PSA of 165 ${Å}^{2}$, came closest to this ideal PSA range. Additionally, dcSTX and dcNeoSTX violated Lipinski’s Rule of Five only once, showing good predicted bioavailability and possessing more negative log Kp values, indicating enhanced skin permeability. Furthermore, they act as substrates for the P-Gp protein. Taken together, these descriptors collectively identify dcSTX and dcNeoSTX derivatives as the most promising candidates with drug-like properties in our study.

## Figures and Tables

**Table 1:** (a) Names, abbreviations, molecular formulae, molecular structures, and LD50 and TEF values of the carbamate division of saxitoxin derivatives. (b) Names, abbreviations, molecular formulae, molecular structures, and LD50 and TEF values of the N-sulfocarbamoyl division of saxitoxin derivatives. (c) Names, abbreviations, molecular formulae, molecular structures, and LD50 and TEF values of the decarbamoyl division of saxitoxin derivatives.

Name	Abbreviation	Molecular Formula	Molecular Structure	LD50	TEF
**(a)**					
Saxitoxin	STX	C_10_H_17_N_7_O_4_	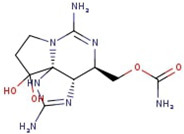	1	1.0
Neosaxitoxin	NeoSTX	C_10_H_17_N_7_O_5_	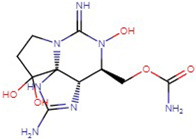	2.54	2.0
Gonyautoxin	GTX1	C_10_H_17_N_7_O_9_S	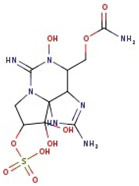	0.93	1.0
Gonyautoxin II	GTX2	C_10_H_17_N_7_O_8_S	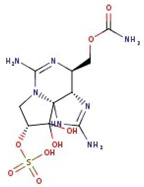	0.57	0.4
Gonyautoxin III	GTX3	C_10_H_17_N_7_O_9_S	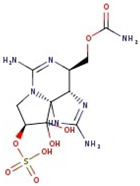	—	0.6
Gonyautoxin IV	GTX4	C_10_H_17_N_7_O_7_S	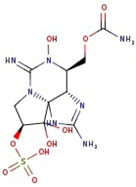	—	0.7
**(b)**					
Gonyautoxin V	GTX5 (B1)	C_10_H_17_N_7_O_8_S	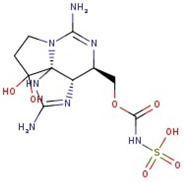	0.064	0.1
Gonyautoxin VI	GTX6 (B2)	C_10_H_17_N_7_O_11_S	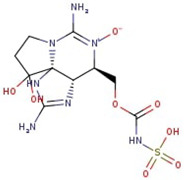	<0.017	0.05
Protogonyautoxin I	C1	C_10_H_17_N_7_O_11_S_2_	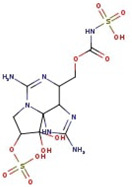	0.043	0.01
Protogonyautoxin II	C2	C_10_H_17_N_7_O_11_S_2_	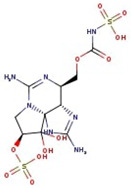	—	0.01
Protogonyautoxin III	C3	C_10_H_17_N_7_O_12_S_2_	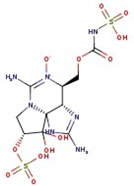	—	0.01
Protogonyautoxin IV	C4	C_10_H_17_N_7_O_12_S_2_	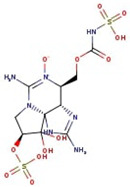	—	0.1
**(c)**					
Decarbamoyl saxitoxin	dcSTX	C_9_H_18_N_6_O_3_	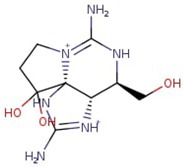	0.37	0.5
Decarbamoyl neosaxitoxin	dcNeoSTX	C_9_H_18_N_6_O_4_	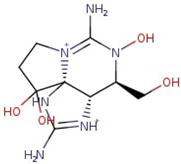	0.22	0.2
Decarbamoyl gonyautoxin	dcGTX1	C_9_H_20_N_6_O_7_S	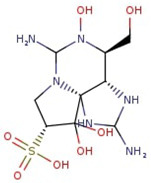	—	—
Decarbamoyl gonyautoxin II	dcGTX2	C_10_H_17_N_7_O_12_S	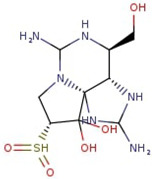	0.11	0.2
Decarbamoyl gonyautoxin III	dcGTX3	C_9_H_20_N_6_O_6_S	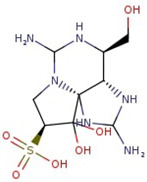	—	0.4
Decarbamoyl gonyautoxin IV	dcGTX4	C_9_H_20_N_6_O_7_S	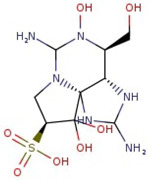	—	—

**Table 2:** Global reactivity descriptors: electron affinity (A), ionization potential (I), electronegativity (χ), hardness (η), electrophilicity (ω) (all in eV), softness (S) (in eV^−1^), nucleophilicity (N), electrodonating power ($\omega^{-}$), electroaccepting power ($\omega^{+}$), and net electrophilicity ($\Delta \omega^{\pm }$) (also in eV) of saxitoxin and its derivatives.

Molecule	A	I	χ	η	ω	S	N	$\omega^{-}$	$\omega^{+}$	$\Delta \omega^{\pm }$
STX	0.32	6.19	3.25	5.87	0.90	0.17	2.61	4.33	0.54	4.89
NeoSTX	0.27	6.26	3.27	5.99	0.89	0.17	2.53	3.79	0.52	4.31
GTX1	0.59	6.29	3.44	5.70	1.04	0.18	2.50	4.16	0.71	4.87
GTX2	0.59	4.88	2.73	4.29	0.87	0.23	3.92	3.38	0.65	4.02
GTX3	−0.05	6.04	2.99	6.10	0.74	0.16	2.75	3.35	0.35	3.70
GTX4	−0.05	6.00	2.97	6.05	0.73	0.17	2.79	3.33	0.35	3.70
GTX5 (B1)	−0.13	5.87	2.87	5.99	0.69	0.17	2.92	3.19	0.32	3.51
GTX6 (B2)	0.30	5.98	3.14	5.68	0.87	0.18	2.81	3.66	0.52	4.18
C1	0.68	6.22	3.45	5.53	1.07	0.18	2.58	4.22	0.77	4.99
C2	0.48	6.29	3.36	5.81	0.99	0.17	2.51	4.03	0.64	4.67
C3	0.55	6.26	3.41	5.72	1.02	0.17	2.53	4.09	0.68	4.77
C4	0.47	6.30	3.39	5.82	0.98	0.17	2.50	4.03	0.64	4.67
dcSTX	0.18	6.00	3.09	5.82	0.82	0.17	2.80	3.54	0.46	4.00
dcNeoSTX	0.27	6.31	3.29	6.05	0.89	0.17	2.48	3.81	0.52	4.33
dcGTX1	0.45	6.34	3.39	5.88	0.98	0.17	2.46	4.02	0.63	4.65
dcGTX2	0.56	6.18	3.77	5.61	1.01	0.18	2.61	4.06	0.69	4.75
dcGTX3	0.59	6.18	3.38	5.60	1.02	0.18	2.61	4.09	0.70	4.79
dcGTX4	0.45	6.34	3.40	5.88	0.98	0.17	2.46	4.02	0.63	4.65

**Table 3:** Drug-likeness properties of saxitoxin and its derivatives (PSA in ${Å}^{2}$, molecular volume in ${Å}^{3}$).

Molecule	Log P	PSA	Molecular Volume	Rule of Five	Rotatable Bonds
STX	−5.04	188	248	2	3
NeoSTX	−3.03	104	255	2	3
GTX1	−5.19	257	304	2	5
GTX2	−5.20	248	295	2	5
GTX3	−5.20	245	295	2	5
GTX4	−5.19	257	304	2	5
GTX5 (B1)	−4.98	225	287	2	4
GTX6 (B2)	−5.44	242	296	2	4
C1	−5.78	289	336	2	6
C2	−5.78	288	336	2	6
C3	−6.01	305	344	2	6
C4	−6.01	305	344	2	6
dcSTX	−6.03	154	221	1	1
dcNeoSTX	−6.04	165	230	1	1
dcGTX1	−5.34	218	275	2	2
dcGTX2	−4.48	186	262	2	2
dcGTX3	−5.32	207	267	2	2
dcGTX4	−5.34	218	275	2	2

**Table 4:** Pharmacokinetics descriptors of saxitoxin and its derivatives.

Molecule	GIA	BBBP	PGPS	I1	I2	I3	I4	I5	Log Kp
STX	Low	No	No	No	No	No	No	No	−11.41
NeoSTX	Low	No	No	No	No	No	No	No	−11.29
GTX1	Low	No	No	No	No	No	No	No	−12.84
GTX2	Low	No	No	No	No	No	No	No	−12.96
GTX3	Low	No	No	No	No	No	No	No	−12.96
GTX4	Low	No	No	No	No	No	No	No	−12.84
GTX5 (B1)	Low	No	No	No	No	No	No	No	−12.27
GTX6 (B2)	Low	No	Yes	No	No	No	No	No	−13.23
C1	Low	No	Yes	No	No	No	No	No	−13.82
C2	Low	No	Yes	No	No	No	No	No	−13.82
C3	Low	No	Yes	No	No	No	No	No	−14.78
C4	Low	No	Yes	No	No	No	No	No	−14.78
dcSTX	Low	No	Yes	No	No	No	No	No	−11.40
dcNeoSTX	Low	No	Yes	No	No	No	No	No	−11.32
dcGTX1	Low	No	Yes	No	No	No	No	No	−13.24
dcGTX2	Low	No	Yes	No	No	No	No	No	−11.25
dcGTX3	Low	No	Yes	No	No	No	No	No	−13.52
dcGTX4	Low	No	Yes	No	No	No	No	No	−13.24

GIA: gastrointestinal absorption; BBBP: brain–blood barrier permeant; PGPS: P-gp substrate; I1: CYP1A2 inhibitor; I2: CYP2C19 inhibitor; I3: CYP2C9 inhibitor; I4: CYP2D6 inhibitor; I5: CYP3A4 inhibitor; log Kp: skin permeation.

## Data Availability

Data are contained within the article.
